# Editorial: Comprehensive Risk Prediction in Cardiomyopathies: New Genetic and Imaging Markers of Risk

**DOI:** 10.3389/fcvm.2022.849882

**Published:** 2022-03-08

**Authors:** Luis Rocha Lopes, Giovanni Quarta, Nuno Cardim, Juan Ramon Gimeno

**Affiliations:** ^1^Barts Heart Centre, St. Bartholomew's Hospital, London, United Kingdom; ^2^Institute of Cardiovascular Science, University College London, London, United Kingdom; ^3^Papa Giovanni XXIII Hospital, Bergamo, Italy; ^4^Hospital da Luz, Lisbon, Portugal; ^5^Universidade Nova de Lisboa, Lisbon, Portugal; ^6^Unidad Centros, Servicios y Unidades de Referencia/European Reference Networks Cardiopatías Familiares, Hospital Clínico Universitario Virgen Arrixaca, Murcia, Spain

**Keywords:** risk, cardiomyopathies, imaging, genetics, strain, cardiac magnetic resonance (CMR), cardiac computed tomographic (CT) imaging, histology

Hypertrophic, (HCM), dilated (DCM), and arrhythmogenic cardiomyopathies (ACM) are major causes of heart failure and sudden death (1). Current management guidelines recommend the use of risk stratification algorithms and more recently scores, to help with important decisions regarding medication initiation/uptitration and device use, including implantable cardioverter-defibrillators or cardiac resynchronization therapy (2–5). These algorithms and scores are mainly comprised of symptomatic status and a few imaging and/or ambulatory ECG markers.

A significant proportion of heart muscle disease is genetically caused and a pathogenic/likely pathogenic variant can be found in around 40–50% of index cases (1). Advances in genetics have allowed for an increasing diagnostic certainty and optimized family screening processes. An influence of genetics in prognosis and outcomes has also been reported in the last few years, but it is yet to be integrated into decision-making recommendations (6).

Great advances in cardiac imaging have also been described in the context of heart muscle disease, including myocardial deformation techniques, scar imaging, perfusion imaging, and tissue characterization (7). These have provided new insights regarding previously unknown phenotypes, including early disease, and already help with differential diagnosis dilemmas in the daily clinical practice. However, unlike old markers such as ejection fraction and wall thickness, these new imaging parameters have not yet been fully integrated in risk prediction algorithms, despite a number of publications describing associations with events (8).

The aim of this Research Topic was to gather contributions from researchers working in the fields of cardiomyopathy genetics and/or cardiomyopathy imaging, who have an interest in establishing a role for new genetics and imaging markers in comprehensive risk prediction in cardiomyopathies.

Segura-Rodriguez et al. described an association between epicardial global longitudinal strain (GLS), non-sustained ventricular tachycardia (NSVT), and late gadolinium enhancement (LGE) on cardiac magnetic resonance in 45 ACM patients, suggesting that a layer-specific GLS approach might offer better acuity compared to overall GLS.

In another paper focusing on myocardial deformation, Kim et al. assessed GLS in 300 patients after pacemaker implantation and found that lower LGS was associated with cardiovascular death and hospitalization for heart failure at 44 ± 28 months follow-up.

In a different and original approach, Bueno-Beti and Asimaki described novel markers of ACM in surrogate tissues, such as the blood and the buccal epithelium, which may represent a non-invasive, safe and inexpensive alternative for diagnosis and cascade screening.

Also on the theme of ACM, Martinez-Sole et al. conducted a very timely systematic review of the exercise influence on the phenotype, concluding that sports may accelerate ACM phenotype either with structural and/or arrhythmic features, and restriction may soften the progression; data on phenotype-negative mutation carriers was also explored, as well as gaps in the current evidence.

Lopez-Ayala et al. analyzed 42 genotype positive-phenotype negative ACM carriers and found that a flattened ST segment may be an early sign of electrical remodeling that precedes T- wave inversion in healthy genetic carriers.

Conte et al. focused their contribution on a less explored imaging modality in the context of cardiomyopathies – when compared to magnetic resonance or echocardiography – by reviewing novel applications of cardiac computed tomography (CT) for a newly diagnosed cardiomyopathy, mainly DCM, including coronary imaging, function assessment, and tissue characterization, but also novel CT applications in HCM and ACM.

Two other articles addressed HCM. Qin et al. described a potential incremental value of T1 mapping to predicted SCD in addition to current guidelines.

An article by Aguiar Rosa et al. described that ischemia was associated with morphological markers of severity of disease, fibrosis, arrhythmia, and functional capacity.

Finally, in a non-genetic heart muscle disease context, Arcari et al. reviewed strain and CMR tissue characterization mapping parameters that have shown recent promise as prognostic predictors for takotsubo syndrome and reviewed the current limited evidence for a possible genetic predisposition.

Overall, the several manuscripts published in this Research Topic demonstrate some of the current trends on cardiomyopathies research: the promising prognostic value of myocardial deformation imaging and tissue characterization for ACM and HCM; the role of myocardial ischemia as a new imaging risk marker in HCM; the value of CT in the diagnosis of cardiomyopathies; new diagnostic approaches for family screening based on the ECG and buccal epithelium for ACM; and the impact of exercise in ACM, including mutation carriers.

## Author Contributions

LL drafted the editorial and approved the final content. All authors reviewed the drafted editorial and approved the final version.

## Conflict of Interest

The authors declare that the research was conducted in the absence of any commercial or financial relationships that could be construed as a potential conflict of interest.

## Publisher's Note

All claims expressed in this article are solely those of the authors and do not necessarily represent those of their affiliated organizations, or those of the publisher, the editors and the reviewers. Any product that may be evaluated in this article, or claim that may be made by its manufacturer, is not guaranteed or endorsed by the publisher.
